# Supplementary material for: An open-source framework for large-scale, flexible evaluation of biomedical text mining systems
Source: J Biomed Discov Collab. 2008 Jan 29;3:1. doi: 10.1186/1747-5333-3-1 (PMC2276192; doi:10.1186/1747-5333-3-1)
Supplement: Additional file 1 — Evaluation platform source code. The additional file contains the software evaluation framework discussed in this paper. The most current release can be downloaded from SourceForge.net [1]. See the accompanying README file for instructions on installation and use. Also included in the distribution is a collection of UIMA wrappers for some commonly used BioNLP tools and annotated corpora. [file 1747-5333-3-1-S1.zip › bionlp_uima_repository-1.0.1/docs/api/edu/uchsc/ccp/uima/ae/util/converter/AnnotationSetOverrider_AE.html]

AnnotationSetOverrider\_AE (Center for Computational Pharmacology Util/UIMA API)


|  |  |  |
| --- | --- | --- |
| **Overview** \| **Package** \| **Class** \| **Tree** \| **Deprecated** \| **Index** \| **Help** | |  |
| PREV CLASS   **NEXT CLASS** | **FRAMES**    **NO FRAMES**     **All Classes** |  |
| SUMMARY: NESTED \| FIELD \| CONSTR \| METHOD | DETAIL: FIELD \| CONSTR \| METHOD |  |


---


## edu.uchsc.ccp.uima.ae.util.converter Class AnnotationSetOverrider\_AE

```
java.lang.Object
  org.apache.uima.analysis_engine.annotator.Annotator_ImplBase
      org.apache.uima.analysis_engine.annotator.JTextAnnotator_ImplBase
          edu.uchsc.ccp.uima.ae.util.converter.AnnotationSetOverrider_AE
```

**All Implemented Interfaces:**: org.apache.uima.analysis\_engine.annotator.BaseAnnotator, org.apache.uima.analysis\_engine.annotator.JTextAnnotator

---

``` public class AnnotationSetOverrider_AE extends org.apache.uima.analysis_engine.annotator.JTextAnnotator_ImplBase ```

Changes annotation set references unless, the annotation has a set in the annotation sets to ignore

**Author:**
:   williamb

---

| **Field Summary** | |
| --- | --- |
| `protected  Set<Integer>` | `annotationSetsToIgnore` |
| `String` | `PARAM_ANNOTATION_SETS_TO_IGNORE` |
| `static String` | `PARAM_SETDESCRIPTION` |
| `static String` | `PARAM_SETID` |
| `static String` | `PARAM_SETNAME` |


**Constructor Summary**

`AnnotationSetOverrider_AE()`


| **Method Summary** | |
| --- | --- |
| `protected  boolean` | `checkForIgnoreBasedOnAnnotationSet(CCPTextAnnotation ccpTA)` |
| `void` | `initialize(org.apache.uima.analysis_engine.annotator.AnnotatorContext ac)` |
| `void` | `process(org.apache.uima.jcas.JCas jcas, org.apache.uima.analysis_engine.ResultSpecification arg1)`             cycle through all annotations and set the annotation set |

**Methods inherited from class org.apache.uima.analysis\_engine.annotator.Annotator\_ImplBase**

`destroy, finalize, getContext, getTypeSystem, reconfigure, typeSystemInit`

**Methods inherited from class java.lang.Object**

`clone, equals, getClass, hashCode, notify, notifyAll, toString, wait, wait, wait`

**Methods inherited from interface org.apache.uima.analysis\_engine.annotator.BaseAnnotator**

`destroy, reconfigure, typeSystemInit`

**Field Detail**

### PARAM\_SETID

```
public static final String PARAM_SETID
```

**See Also:**: Constant Field Values

---


### PARAM\_SETNAME

```
public static final String PARAM_SETNAME
```

**See Also:**: Constant Field Values

---


### PARAM\_SETDESCRIPTION

```
public static final String PARAM_SETDESCRIPTION
```

**See Also:**: Constant Field Values

---


### PARAM\_ANNOTATION\_SETS\_TO\_IGNORE

```
public final String PARAM_ANNOTATION_SETS_TO_IGNORE
```

**See Also:**: Constant Field Values

---


### annotationSetsToIgnore

```
protected Set<Integer> annotationSetsToIgnore
```


**Constructor Detail**

### AnnotationSetOverrider\_AE

```
public AnnotationSetOverrider_AE()
```


**Method Detail**

### initialize

```
public void initialize(org.apache.uima.analysis_engine.annotator.AnnotatorContext ac)
                throws org.apache.uima.analysis_engine.annotator.AnnotatorInitializationException,
                       org.apache.uima.analysis_engine.annotator.AnnotatorConfigurationException
```

:   **Specified by:**: `initialize` in interface `org.apache.uima.analysis_engine.annotator.BaseAnnotator` **Overrides:**: `initialize` in class `org.apache.uima.analysis_engine.annotator.Annotator_ImplBase`
:   **Throws:**: `org.apache.uima.analysis_engine.annotator.AnnotatorInitializationException`: `org.apache.uima.analysis_engine.annotator.AnnotatorConfigurationException`

---


### process

```
public void process(org.apache.uima.jcas.JCas jcas,
                    org.apache.uima.analysis_engine.ResultSpecification arg1)
             throws org.apache.uima.analysis_engine.annotator.AnnotatorProcessException
```

:   cycle through all annotations and set the annotation set

    :   **Throws:**: `org.apache.uima.analysis_engine.annotator.AnnotatorProcessException`

---


### checkForIgnoreBasedOnAnnotationSet

```
protected boolean checkForIgnoreBasedOnAnnotationSet(CCPTextAnnotation ccpTA)
```


---


|  |  |  |
| --- | --- | --- |
| **Overview** \| **Package** \| **Class** \| **Tree** \| **Deprecated** \| **Index** \| **Help** | |  |
| PREV CLASS   **NEXT CLASS** | **FRAMES**    **NO FRAMES**     **All Classes** |  |
| SUMMARY: NESTED \| FIELD \| CONSTR \| METHOD | DETAIL: FIELD \| CONSTR \| METHOD |  |


---
